# Adsorption Hysteresis in Open Slit-like Micropores

**DOI:** 10.3390/molecules26165074

**Published:** 2021-08-21

**Authors:** Grygorii Dragan, Volodymyr Kutarov, Eva Schieferstein, Alexander Iorgov

**Affiliations:** 1 Physics Research Institute, Odesa I.I. Mechnikov National University, 27 Pasteur St., 65082 Odesa, Ukraine; dragan@onu.edu.ua (G.D.); v.kutarov@onu.edu.ua (V.K.); a.iorgov@onu.edu.ua (A.I.); 2 Fraunhofer UMSICHT, 3 Osterfelder Str., D-46047 Oberhausen, Germany

**Keywords:** adsorption, desorption, hysteresis, modelling, fractal dimension, degree of irreversibility

## Abstract

Adsorption hysteresis in the low-pressure range is only rarely described in the literature. To optimise, for example, heat storage technologies, a deeper understanding of the low-pressure hysteresis (LPH) process is necessary. Here, two thermodynamically based approaches are further developed for analysing the LPH within the framework of thermodynamically irreversible processes and fractal geometry. With both methods developed, it is possible to obtain the description of the adsorption and desorption branches with high accuracy. Within the framework of the two thermodynamic models of the hysteresis loop, generalised equations are obtained with the control parameter in the form of the degree of irreversibility. This is done by taking the adsorption of water on alumina as an example. It is shown that the fractal dimension of the adsorption process is larger than the fractal dimension of the desorption branch, meaning that the phase state of the adsorbate is more symmetric during the adsorption step than in the desorption process.

## 1. Introduction

In recent years, it has become apparent that the only way to avoid irreversible climate change and the dramatic effects of global warming on the planet is to reduce carbon dioxide emissions. This can be achieved through the introduction of more efficient energy systems and the use of energy sources that do not allow CO_2_ emissions.

To solve the problem of global warming, an important role is played by heat storage technologies, which are based on adsorption processes. In this case, thermal energy is accumulated in the form of adsorption heat and is released during desorption. The processes of water vapor adsorption–desorption are the most promising for the accumulation of thermal energy. Since these adsorption systems often show hysteresis, a theoretical study of adsorption hysteresis is of great interest [1].

The types of hysteresis loops are classified in the IUPAC Technical Report [2], but only for hysteresis at large relative pressures. The existence of low-pressure hysteresis (LPH) is mentioned and attributed only to non-rigid solids with micropores, where LPH is explained by the swelling of the non-rigid porous structure.

One interesting type in the IUPAC classification is the H3 loop, which is often shown when “the adsorption branch resembles a Type II isotherm”. As shown by Gregg and Sing, a hysteresis loop which resembles the H3 loop very often continues into the low-pressure region, and often does not close, even at $p/p_{0}\to 0\;$[3].

More than half a century ago, Arnell and McDermot suggested that hysteresis at low pressures is due to the formation of “traps” in the adsorbent during adsorption, and that adsorbate molecules falling into these traps either desorb very slowly or do not desorb at all [4,5]. “Traps” in the adsorbent can be of two types: diffusional and chemical. Diffusional traps arise because a deformation of the adsorbent takes place during the adsorption. This means that cavities that were previously inaccessible to adsorbate molecules open, allowing access. Due to irreversible changes in the framework of the adsorbent, the structure of the sorbent changes during desorption, which significantly slows the diffusion of the adsorbate from the cavities of the adsorbent into the environment. Chemical traps arise due to the formation of bonds between polar adsorbate molecules and exchangeable adsorbent cations. A typical example is the formation of chemical traps in layered silicates. The formation of two types of traps leads to the potential barrier to desorption increasing in comparison with the potential barrier to adsorption.

It can be argued that the nature of adsorption hysteresis in the region of micropores is generally well-understood. In addition, various methods for describing adsorption isotherms in micropores have been extensively studied [3,6]. However, theoretical methods for the analysis of adsorption hysteresis in micropores are insufficiently presented.

A thermodynamically grounded approach to the analysis of adsorption hysteresis is the method of cycles [6,7,8].

Adsorption hysteresis in the set of micropores is characterised by certain features associated with the state of the adsorbate in the pores of the adsorbent. In the set of micropores, the adsorbed phase is defined as a quasi-one-dimensional (1D) phase. In the quasi-1D phase, a first-order phase transition is impossible [9].

When analysing adsorption hysteresis in the set of micropores, the adsorption–desorption transition should be defined as a disorder–order transition. In this case, adsorption is characterized as a short-range order process, and desorption as a long-range order process [3]. Thus, adsorption hysteresis should be considered as a change in the degree of symmetry of the adsorbed phase. Fractal geometry is one of the possible approaches to the analysis of changes in the symmetry of the adsorbed phase in the adsorption–desorption processes [10,11].

We previously developed a thermodynamically substantiated method for calculating the adsorption–desorption process and hysteresis loop in the region of micropores.

Two approaches have been proposed for calculating the hysteresis loop, based on:

(1) The classical theory of volume filling in micropores [4];
(2) The alternative theory of adsorption in micropores [5].

The aim of this work is to further develop methods for analysing the adsorption hysteresis loop within the framework of thermodynamically irreversible processes and fractal geometry.

## 2. Methods

### 2.1. The Classical Theory of Adsorption in Micropores

In the following, the H3-type hysteresis loop is considered for slit-like pores. The adsorption process in the area of micropores occurs in pores with a characteristic size *h* in the order of $\left(1.6-1.8\right)\;*\;\sigma =h$ (σ: molecular diameter of the adsorbate) [3]. In channels of this size, the adsorbate behaves as a quasi-one-dimensional phase in the potential field of the pore walls. The theory of the equation of state for a one-dimensional phase in a potential field has been developed quite well. However, this theory is complex, and its practical application is associated with significant mathematical difficulties.

For this reason, in order to describe the equilibrium properties of the adsorbate on the adsorption and desorption branches of the hysteresis loop in the first approximation, in this work we used the theory of volume filling of micropores (TVFM), which has proven itself in practice [3].

The most general TVFM equation is the Dubinin–Astakhov (DA) equation [12,13,14]:

(1) $$\theta =exp\left[-{\left(\frac{A}{\varepsilon }\right)}^{\alpha }\right]\;\;\;\;\;\;\to \;\;\;\;\;\ln\left(-\ln\theta \right)=\alpha *\;\left(\ln A-\ln\varepsilon \right)$$

The governing parameter in Equation (1) is the adsorption potential (Gibbs potential), $A=-\Delta G=RT\;\ln(p_{0}/p),\;$ where $p_{0}$ and p are the saturation pressure and equilibrium pressure of the adsorbate in the bulk phase at temperature *T*. The parameter *θ* to be determined is $\theta =a/a_{m}$, where a and $a_{m}$ are respectively the adsorption values for some intermediate value of the determining parameter *A* and the value of adsorption for all pores for the right boundary of the micropore region. Equation (1) was obtained under the assumption that the distribution of the determined parameter *θ* over the determining parameter *A* can be represented by the Weibull distribution [15]:

(2a) $$f_{i\;}\left(\frac{A_{i}}{\varepsilon_{i}}\right)=\alpha_{i}\;{\left(\frac{A_{i}}{\varepsilon_{i}}\right)}^{\alpha_{i}-1}*exp\left[-{\left(\frac{A_{i}}{\varepsilon_{i}}\right)}^{\alpha_{i}}\right]$$

(2b) $$f_{1}\left(\frac{A_{1}}{\varepsilon_{1}}\right)=\left(\alpha_{1}*\varepsilon_{1}\right)\frac{A_{1}^{\alpha_{1}-1}}{\varepsilon_{1}^{\alpha_{1}}}*exp\left[-{\left(\frac{A_{1}}{\varepsilon_{1}}\right)}^{\alpha_{1}}\right]$$

Here and in the following, the index values *i* = 1 and 2 refer to adsorption and desorption, respectively.

The coefficients $\alpha_{i}$ and $\varepsilon_{i}$ are the control parameters of the Weibull distribution. The parameter $\varepsilon_{i}$ should be considered as the averaged value of the potential barrier to adsorption $\varepsilon_{1}$ and desorption$\;\varepsilon_{2}$. According to the concept of the nature of adsorption hysteresis in the micropore region, it is possible to determine the relationship between the potential barriers to adsorption and desorption:

(3) $$\varepsilon_{2\;}=\varepsilon_{1}+\Delta \varepsilon$$

where Δε is the difference between the potential barriers of adsorption and desorption.

Equation (1) contains two defining parameters *α* and *ε*. Let us introduce the third control parameter:

(4) $$k=1+\frac{\Delta \varepsilon }{\varepsilon_{1}}$$

Equation (4) determines the degree of irreversibility of the adsorption process *k*. For a reversible adsorption process, Δε=0 and *k* = 1.

Thus, if a hysteresis loop of type H3 is obtained experimentally, Equations (1) and (3) describe the irreversible adsorption in micropores.

The task in this work is the general description of H3 hysteresis loops for microporous systems. Therefore, the determination of the equations describing the adsorption and desorption branches is necessary.

Represented by Equation (2) in the form

(5) $$f_{i}\left(\frac{A_{i}}{\varepsilon_{i}}\right)=\left(\alpha_{i}\varepsilon_{i}\right)\frac{1}{A_{i}}\;{\left(\frac{A_{i}}{\varepsilon_{i}}\right)}^{\alpha_{i}}exp\left[-{\left(\frac{A_{i}}{\varepsilon_{i}}\right)}^{\alpha_{i}}\right],$$

the Weibull distribution has several specific mathematical properties. On the basis of these properties, it can be assumed that within the same thermodynamic cycle the product of the parameters ($\alpha_{i}\varepsilon_{i}$), calculated for the forward and reverse branch of the adsorption–desorption cycle, is constant. These distribution parameters can be represented by the following relationship:

(6) $$\left(\alpha_{1}\varepsilon_{1}\right)=\left(\alpha_{2}\varepsilon_{2}\right)$$

The validity of relation (6) was confirmed on the basis of the analysis of the adsorption and desorption isotherms of various adsorption systems [16].

The potential barrier of desorption is determined according to Equation (3). Then, relation (6) can be rewritten as follows:

(7) $$\alpha_{1}=\alpha_{2}\left(1+\frac{\Delta \varepsilon }{\varepsilon_{1}}\right)$$

Substituting Equation (7) into Equation (1), we obtain the final version of the extended Dubinin–Astakhov equation—the so-called Dubinin–Astakhov-Advanced (DAA) equation—to determine the degree of filling of micropores in the adsorption and desorption process, respectively:

(8) $$\theta_{1}=exp\left[-{\left(\frac{A_{1}}{\varepsilon_{1}}\right)}^{\alpha_{1}}\right]$$

(9) $$\theta_{2}=exp\left[-{\left(\frac{A_{2}}{\varepsilon_{1}k}\right)}^{\alpha_{1}/k}\right]$$

The DAA equation differs by the initial introduction of an additional third control parameter $k=1+\Delta \varepsilon /\varepsilon_{1}$. This option should be regarded as the degree of irreversibility of the adsorption system. If the difference of the potential barriers is zero, the adsorption process in the low-pressure region is reversible k=1 and the Equations (8) and (9) become the original DA Equation (1). Thus, the analytical DAA equation is proposed to describe the H3 hysteresis loop in micropores.

For what follows, we introduce a more general definition of the filling of micropores in the form:

(10) $$\theta =\frac{a_{1,2}-a_{m,1,2}}{a_{0}-a_{m,1,2}}$$

In the definition, $a_{1,2}$ is the amount of adsorbate in micropores for some intermediate value of the determining parameter $A_{1,2}$ during adsorption and desorption, respectively. The value $a_{m,1,2}$ was determined earlier. The amount of $a_{0}$ is proposed to $a_{0}\sim \frac{h}{\sigma }*a_{m}$.

Based on formulas (8) and (9), the following equations can be written for the adsorption and desorption branches of the hysteresis loop:

(11) $$\mathrm{Adsorption}:\;a_{1}=a_{m,1}\left\{1+\left(\frac{h}{\sigma }-1\right)exp\left[-{\left(\frac{A_{1}}{\varepsilon_{1}}\right)}^{\alpha_{1}}\right]\right\}$$

(12) Desorption: a2=am,21+hσ−1exp−A2ε1kα1k

Let us define the desorption loop from the condition

(13) $$a_{2}x_{2}=a_{1}x_{1}$$

Then, the value of the relative pressure on the desorption branch of the hysteresis loop is determined as follows. Let us rewrite Equations (4), (7), and (12) in the form:

(14) A2=ε1k−lna2am,2−1hσ−1−1kα1

The value of the relative pressure of desorption $x_{2}$ is calculated as follows:

(15) $$x_{2}=exp\left(-\frac{A_{2}}{RT}\right)$$

Equations (14) and (15) will further be used to describe the desorption branch of the hysteresis loop.

### 2.2. An Alternative Theory of Adsorption in Micropores

It is assumed that the process of adsorption in micropores occurs in the same way as the volume filling process, which has been studied in other works. TVFM is based on the assumption that the adsorption in the pores should be treated by volume filling (similar to the capillary condensation process), rather than by layer-by-layer filling. The physical analogy between both processes implies their formal analogy, that is, the volume filling of pores and the capillary condensation could be expected to obey a similar mathematical treatment.

For the pore width range $\sigma <h<\left(1.6-1.8\right)\;*\;\sigma$, a quasi-one-dimensional phase (molecular associate, cluster which does not exhibit any surface tension) is formed in the pore.

The capillary condensation process is described by the Kelvin equation [13]:

(16) $$x=\frac{p}{p_{0}}=exp\left(-\frac{\lambda \;V_{l}}{RT\;t\;\sigma }\right)$$

Here, λ and $V_{l}$ are the surface tension and molar volume of liquid adsorbate, σ is the van der Waals’ diameter of the adsorbate molecule, and *t* is the thickness of the condensate film on the pore walls. The governing parameter in Equation (16) is the relationship between capillary and adsorption forces [17,18].

In particular, the expression analogous to Kelvin’s equation was proposed where the governing parameter φ is the molecular associate energy in the potential field of pore walls. For slit-like pores, the equation derived in [17] becomes:

(17) $$x=\frac{p}{p_{0}}=exp\left\{\frac{\varphi z_{0}}{RT\;h}\left[1-\frac{1}{2h}\left(\chi -1\right)\right]\right\}$$

where *h* is the pore width, $z_{0}$ and *χ* (0≤χ≤1) determine the geometric characteristics of the pore space, and φ is the potential energy of interaction of molecules in a potential field of walls. In [17], the parameter $z_{0}$ is rigorously and analytically determined, but in practical applications its calculation is too complicated.

On the other hand, within the transition region the approximation $h/z_{0}\approx h/\sigma =\theta$ provides reasonable accuracy for low-molecular-weight substances.

The potential energy of interaction of molecules in a potential field of walls is [4]

(18) $$\varphi =\varphi_{0}\frac{h/\sigma }{h/\sigma -B}$$

where $\varphi_{0}$ is the potential of interaction of molecules for h/σ → ∞.

We give a brief analysis of Equation (17). In general, it is not possible to consider the influence of the parameter *χ* in Equation (17). Consider two limiting cases of the geometric characteristics of the pore space [17]:

(19) $$x=\frac{p}{p_{0}}=exp\left\{\frac{\varphi }{RT\theta }\left[1+\frac{1}{2h}\right]\right\}\;\;\mathrm{for}\;\chi =0$$

(20) $$x=\frac{p}{p_{0}}=exp\left\{\frac{\varphi }{RT\theta }\right\}\;\;\mathrm{for}\;\chi =1$$

However, in the framework of the thermodynamics of disordered media, the exponent in formula (17) can be represented in the following form:

(21) $$x=exp\left[{\left(\frac{\varphi }{RT\theta }\right)}^{\kappa }\right]$$

For *χ* = 1 is *κ* = 1; for *χ* = 0 is *κ* < 1.

For further calculations, we represent formula (21) in the form:

(22) RTlnx1κ=φθ

Introducing the adsorption potential $A=RT\ln\left(1/x\right)$, Equation (23) is obtained from Equation (22) [5]:

(23) $$\theta =B+{\left(\frac{A_{0}}{A}\right)}^{1/\kappa }$$

Here $B=\theta_{0}$ is the initial filling of micropores determined by formula (23) and $A_{0}$ is the adsorption potential corresponding to the relative pore range where the influence of the opposite pore walls becomes negligibly small—that is,$\;A_{0}/RT=\varphi_{0}/RT=-\ln\left(x_{0}\right)$, where $x_{0}$ is the relative pressure at which $\bar{\varphi }\to 1$. Therefore, Equation (23) is obtained here in the framework of the basic TVFM postulate.

Let us now consider the description of the hysteresis loop based on Equation (23). We write Equation (23) in the form:

(24) $$\theta_{1,2}=B_{1,2}+{\left(\frac{A_{1,2}^{0}}{A_{1,2}}\right)}^{\beta_{1,2}}$$

where $\beta_{1,2}=1/\kappa_{1,2}$ indices 1 and 2 refer, as before, to adsorption and desorption processes, respectively. Let us introduce by analogy with formulas (3) and (4) the value of the potential barrier for the processes of adsorption $\Delta A_{1,2}^{0}$ and desorption, and the irreversibility parameter *k*.

(25) $$\Delta A_{1,2}^{0}=A_{2}^{0}-A_{1}^{0}$$

(26) $$k=1+\frac{\Delta A_{1,2}^{0}}{A_{1}^{0}}$$

Let us write Equation (24) for the hysteresis loop:

(27) $$\mathrm{Adsorption}:\;a_{1}=a_{m,1}\left[B_{1}+{\left(\frac{A_{1}^{0}}{A_{1}}\right)}^{\beta_{1}}\right]$$

(28) $$\mathrm{Desorption}:\;a_{2}=a_{m,2}\left[B_{2}+{\left(\frac{A_{2}^{0}}{A_{2}}\right)}^{\beta_{2}}\right]$$

Based on formulas (13), (27), and (28), and under the following assumptions:

(29) $${\left(A_{1}^{0}\right)}^{\beta_{1}}={\left(A_{2}^{0}\right)}^{\beta_{2}}\;;\;\beta_{2}=k\beta_{1}\;;\;B_{2}=B_{1}\frac{a_{m,2}}{a_{m,1}}\;;\;A_{1}^{0}\;\beta_{2}=A_{2}^{0}\;\beta_{1}$$

we obtain an equation for describing the desorption branch of the hysteresis loop:

(30) $$A_{2}=kA_{1}^{0}{\left(\frac{a_{1}}{a_{m,2}}-B_{1}\frac{a_{m,2}}{a_{m,1}}\right)}^{-\left(\frac{1}{\beta_{1}k}\right)}$$

The value of the relative pressure of desorption $x_{2}$ is calculated as follows:

(31) $$x_{2}=exp\left(-\frac{A_{2}}{RT}\right)$$

In the future, Equations (29) and (30) will be applied to predict the desorption branch of the hysteresis loop.

### 2.3. Adsorption Hysteresis and Fractal Characteristics in Microporous Structures

Previously, we proposed a method for analysing the fractal characteristics of microporous materials, based on the analytical solution of the integral Dubinin equation [18]. Here are the main provisions of the developed method. The integral equation of M. Dubinin has the following form:

(32) $$\theta \left(T,p\right)=\overset{l_{max}}{\underset{l_{min}}{\int }}f\left(l\right)\;*\;exp\left[-\left(\frac{lA}{k\beta }\right)\right]\;dl$$

Then, Equation (31) should be rewritten as:

(33) $$\theta \left(T,p\right)=\overset{\infty }{\underset{0}{\int }}ml^{3-D}*\;exp\left[-{\left(\frac{lA}{k\beta }\right)}^{2}\right]\;dl$$

(34) $$\theta \left(T,p\right)=\overset{\infty }{\underset{0}{\int }}\varphi \left(l\right)\;*\;l^{3}*exp\left[-{\left(\frac{lA}{k\beta }\right)}^{2}\right]dl\;;\;with\;\varphi \left(l\right)=m*l^{-D}\;$$

For analytical solutions of Equation (33) we make the following additional assumptions:

(1) The quantity $\theta \left(T,p\right)$ is an additive function;
(2) In Equation (31), the integrand has a sharp maximum. This determines the maximum value of $\theta \left(T,p\right)$ corresponding to a certain $l^{*}(l_{min}<l^{*}<l_{max})$ for a given value of *A*;
(3) The function of density distribution $\varphi \left(l\right)=ml^{-D}$ is a continuous and slowly varying function;
(4) In the neighbourhood of $l^{*}$ the function f (*l*) has a constant value equal to the value f ($l^{*}$).

Therefore, to solve Equation (33) one can apply the method of steepest descent, also known as the “saddle point” method. Assuming that for the maximum of the integrand, in the vicinity of $l^{*}$, the function $\varphi \left(l\right)$ takes a constant value of $\varphi \left(l^{*}\right)$, Equation (33) can be rewritten as follows [15]:

(35) $$\theta \left(T,p\right)=\varphi \left(l^{*}\right)\overset{\infty }{\underset{0}{\int }}l^{3}*exp\left[-{\left(\frac{lA}{k\beta }\right)}^{2}\right]dl$$

Equating the derivative of the integrand to zero, one can determine the value $l^{*}$:

(36) l∗=32∗kβA 

After this, integrating (34), we obtain:

(37) $$\theta \left(T,p\right)=\frac{1}{2}{\left(\frac{k\beta }{A}\right)}^{4}\varphi (l^{*})$$

From Equation (36) we determine the formula for determining the distribution function $\varphi \left(l^{*}\right)$:

(38) $$\varphi \left(l^{*}\right)=2\theta \left(T,p\right){\left(\frac{A}{k\beta }\right)}^{4}$$

As stated above, the distribution function is defined as $\varphi \left(l\right)=ml^{-D}$.

Thus, on the basis of formula (37) and with the experimental values $\theta \left(T,p\right)$ and *A,* one can determine the fractal dimension *D* as follows:

(39) $$2\theta \left(T,p\right){\left(\frac{A}{k\beta }\right)}^{4}=m*{\left(l^{*}\right)}^{-D}$$

(40) $$\ln\left[2\theta \left(T,p\right){\left(\frac{A}{k\beta }\right)}^{4}\right]=\ln\varphi \left(l^{*}\right)=\ln m-D*\ln\left(l^{*}\right)$$

To analyse the experimental data, we represent formula (38) with the help of Equation (35) in the form:

(41) $$\theta \left(T,p\right)=A^{D-4}\;\;\;\;\Leftrightarrow \;\;\;\ln\theta \left(T,p\right)=\left(D-4\right)\ln A$$

## 3. Results and Discussion

**Figure 1:**
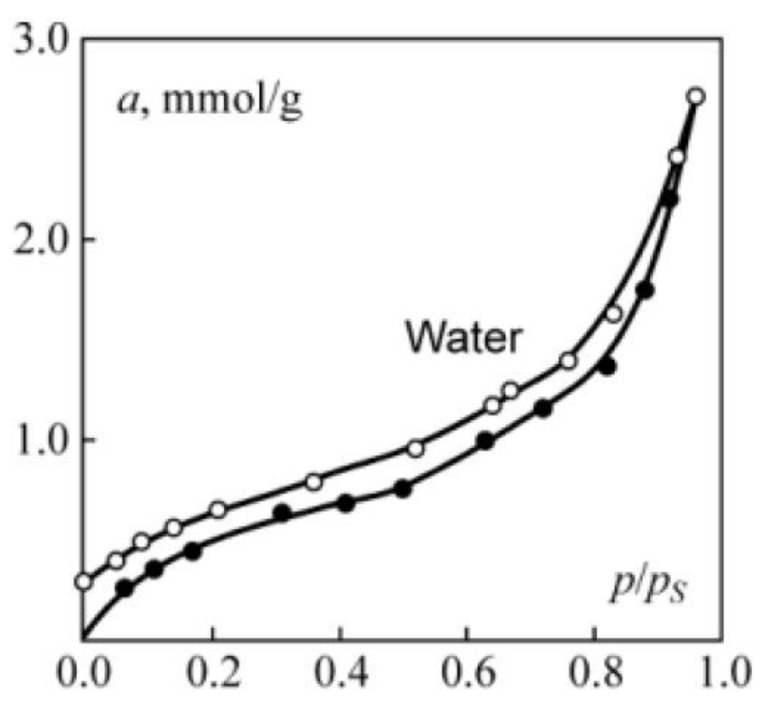
Isotherms of adsorption (●) and desorption (○) of water vapor on Al_2_O_3_.

As an example, let us consider the adsorption–desorption isotherm of water vapor on an Al_2_O_3_ sample (Figure 1). A preliminary analysis of the adsorption isotherm (Figure 1) was carried out on the basis of the t-plot [3].

**Figure 2:**
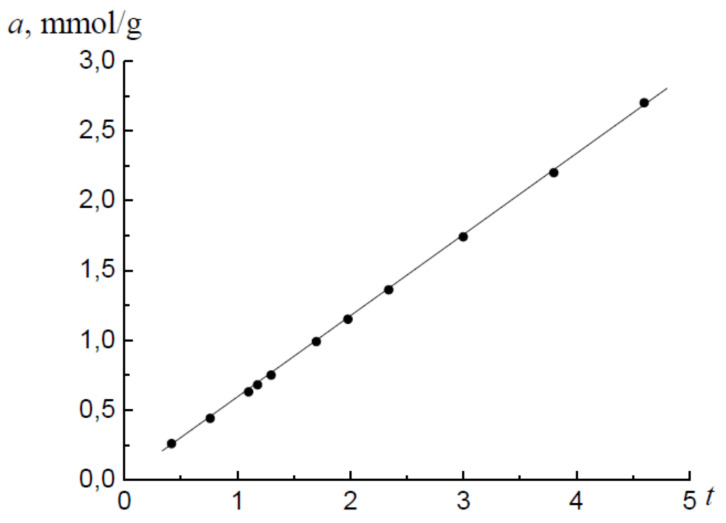
The t-plot for the adsorption branch of the isotherm of water vapor on Al_2_O_3_.

Figure 2 shows the isotherm of water vapor adsorption on the Al_2_O_3_ sample in coordinates $a=f\left(t\right),\;$where $t=a/a_{m}$ is the statistical thickness of the monolayer. For further analysis, the adsorption values of the monolayer were determined by the BET method [3]. For the adsorption isotherm $a_{m,1}=0.61$ mmol/g, for the desorption isotherm, $a_{m,2}=0.63$ mmol/g. The plot shown in Figure 2 is characteristic of the adsorption process in slit-like pores. The condition for determining the area of micropores is 1<h/σ≤1.6−1.8 [3]. For definiteness, let us choose a value for the right boundary of the micropore region to h/σ=1.7. Then, for the adsorption values of the right boundary micropores on the adsorption branch, it is valid that $0.61<a_{1}\leq 1.0$ mmol/g, and for the desorption branch, $0.63<a_{2}\leq 1.07$ mmol/g.

**Figure 3:**
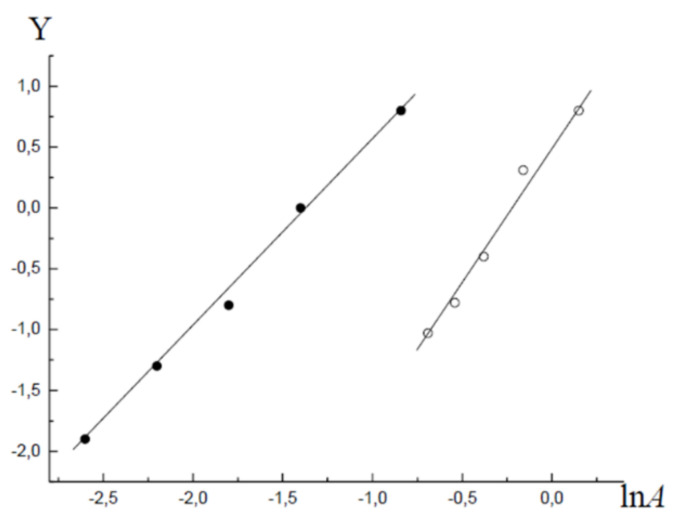
The adsorption (●) and desorption (○) branches of the hysteresis loop in the coordinates of the linearised Equation (1); $Y=\ln\left(-\ln\theta \right)$.

To determine the parameters of Equation (1), we analysed the adsorption and desorption branches of the isotherm hysteresis loop (Figure 1) using the standard method [3]. The analysis results are shown in Figure 3. For the parameters of Equation (1), the following values were obtained: $\varepsilon_{1}=0.66;\;\alpha_{1}=1.95;\;\varepsilon_{2}=0.86;\;\alpha_{2}=1.5$. Equations (8) and (9), with the obtained parameter values, describe the experimental adsorption–desorption isotherm with a maximum relative deviation of ±δ=8.5%.

The numeric values of the parameters $\varepsilon_{1},\;\alpha_{1}\;\mathrm{and}\;\varepsilon_{2},\;\alpha_{2}$ satisfy condition (6). The irreversibility parameter of the hysteresis loop is *k* = 1.3. Note that this value of the parameter *k* coincides, on average, with the values of the parameter *k* for the adsorption systems considered in [16]. Thus, the analysis of adsorption hysteresis was carried out within the framework of the classical theory of adsorption in micropores.

**Figure 4:**
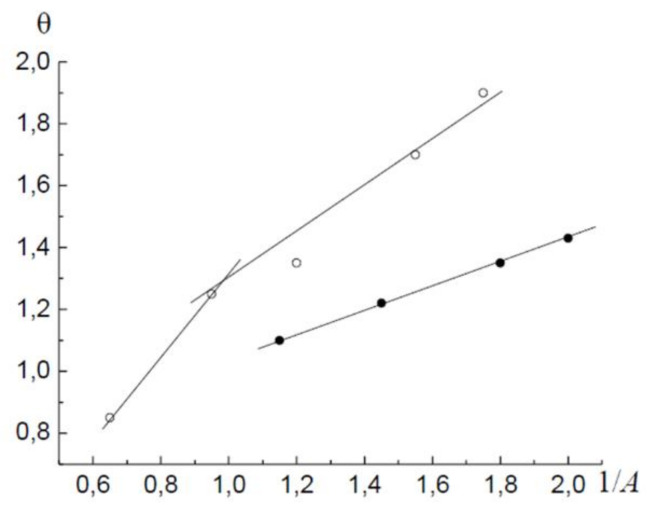
The adsorption (●) and desorption (○) branches of the hysteresis loop in the coordinates of Equation (24).

Now let us analyse the isotherm in Figure 1 on the basis of an alternative theory of the adsorption process in micropores. In Figure 4 the segment of the adsorption isotherm in the micropore region is presented in the coordinates of Equation (24). It is seen that the adsorption branch of the hysteresis loop can be represented by Equation (27), with the parameters $B_{1}=0.77;\;A_{1}^{0}=0.32\;RT;\;\beta_{1}=1$.

**Figure 5:**
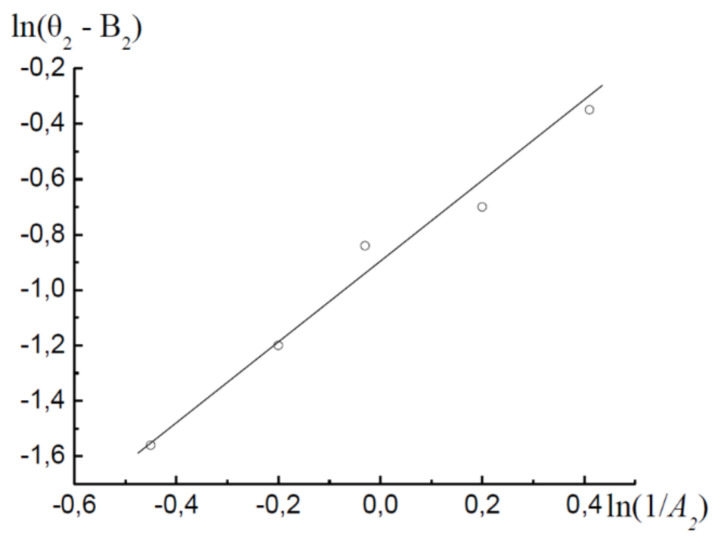
Desorption branch of the hysteresis loop in the coordinates of the linearised Equation (24).

The desorption branch of the hysteresis loop in the region of micropores must be described with a parameter *β* not equal to unity (Figure 5). With Equation (28) for the desorption branch of the hysteresis loop in the region of micropores, the following values were obtained: $B_{2}=0.82;\;A_{2}^{0}=0.42\;RT;\;\beta_{2}=1.23$.

With these values of the parameters, Equation (24) describes the adsorption and desorption branches of the hysteresis loop in the region of micropores, with a maximum relative deviation of ±δ=7.8%.

The irreversibility parameter of the hysteresis loop determined by the alternative theory of adsorption in micropores is *k* = 1.35. The comparison of the irreversibility parameter *k*, calculated in the TVFM formalism and the alternative theory of adsorption in micropores, gives a good coincidence of *k* for both models.

Let us now consider the nature of adsorption irreversibility in micropores within the framework of the fractal geometry formalism.

Adsorption hysteresis in micropores is determined by two factors: the pore size distribution function and the structural state of the adsorbate in micropores.

**Figure 6:**
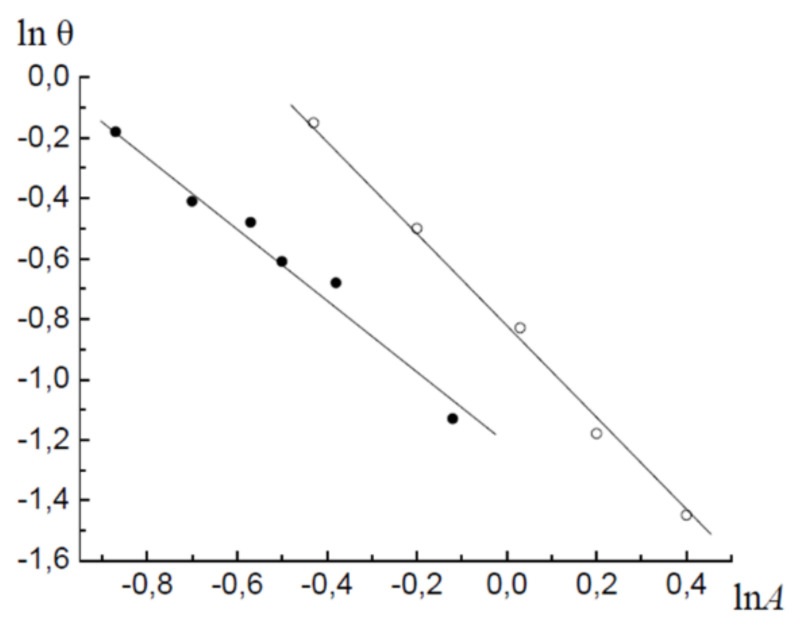
Linearised scaling dependence (40) for the adsorption (●) and desorption (○) branches of the hysteresis loop.

Let us consider the determination of the fractal dimension of a microporous sample on the adsorption and desorption branches of the hysteresis loop. The corresponding calculations were carried out using formulae (34, 41). The calculation results are shown in Figure 6. The following values of the fractal dimension were obtained: for the adsorption process $D_{1}=2.75$, for the desorption process $D_{2}=2.33$. Now let us give a number of explanations about the obtained values of the fractal dimension for the adsorption and desorption branches of the hysteresis loop.

To determine the structural characteristics of a set of pores in the theory of fractal structures, the connectivity index of the set is introduced$\;\mu_{c}\;$[11]:

(42) $$\mu_{c}\;=\;2(D-1)$$

The connectivity index number of the set of pores considered by formula (42) in the process of adsorption is$\;\mu_{c,1}$ = 3.5 and in the process of desorption$\;\mu_{c,2}$ = 2.66. The smaller value of the connectivity index of the set of pores during desorption is determined by the long-range order characteristic of desorption [3].

Now, let us carry out a comparative analysis of the results obtained in the description of the hysteresis loop for the adsorption of water vapor on the Al_2_O_3_ sample, and for the adsorption systems presented in Table 1. The adsorption–desorption processes for all adsorbate–adsorbent systems are characterized by large values of potential barriers compared to potential barriers for the hysteresis loop during the adsorption–desorption of water vapor on the Al_2_O_3_ sample.

Adsorbents such as lunar regolith, shungite, and kaolinite are characterised by a high degree of adsorption deformation (swelling) compared to Al_2_O_3_. Additionally, these adsorbents form stronger covalent bonds with water molecules [19,20,21,22,23,24]. In this case, diffusional and chemical “traps” have higher values in comparison with the values of traps during the adsorption–desorption of water vapor on the Al_2_O_3_ sample.

Note that the parameter *α* for the processes of adsorption–desorption and the degree of irreversibility for the hysteresis loop *k* during the adsorption of water vapor on the lunar regolith and shungite samples are close in value to the analogous parameters for the adsorption of water vapor on the Al_2_O_3_ sample.

Equations (6) and (29) should be considered as thermodynamic invariants of the hysteresis loop during adsorption in micropores.

In the future, a more detailed study of thermodynamic invariants and their relationship with the geometric characteristics of the adsorbate and adsorbent is required.

## 4. Conclusions

The classical theory of volume filling in micropores (DA equation) and the alternative theory of adsorption in micropores (generalised Kelvin equation) are presented in the form of universal equations. The proposed universal equations are based on a thermodynamic invariant. The determination of the thermodynamic invariant is based on the values of potential barriers to adsorption–desorption and the degree of deviation of the pore size distribution function from the Gaussian distribution.

The governing parameter of the universal equations is the degree of irreversibility of the thermodynamic adsorption–desorption cycle.

Thus, it is possible to predict the desorption branch of the hysteresis loop based on the governing parameters for the adsorption branch.

The hysteresis loop in the set of micropores is defined as a disorder–order transition. Higher potential barriers to desorption determine a greater degree of phase order and a lower degree of symmetry with respect to the adsorption phase. This is expressed in a smaller value of the fractal dimension.

## Figures and Tables

**Table 1:** Parameters of the DA Equation (1).

Adsorption System	Adsorption		Desorption		Δε/RT	*k*
	$\Delta \varepsilon_{1}/RT$	$\alpha_{1}$	$\Delta \varepsilon_{2}/RT$	$\alpha_{2}$		
Water–lunar regolith [19]	2.61	1.77	3.75	1.18	1.14	1.44
Water–shungite [20]	4.28	1.62	5.50	1.19	1.22	1.29
Water–kaolinite [21,22]	1.95	2.50	2.14	2.20	0.19	1.10
Pyridine–montmorillonite [23,24]	3.24	1.91	4.69	1.41	1.45	1.45

Water–lunar regolith $\left(\alpha_{1}\varepsilon_{1}\right)$ = 4.61; $\left(\alpha_{2}\varepsilon_{2}\right)$ = 4.41. Water–shungite:$\;\left(\alpha_{1}\varepsilon_{1}\right)$ = 6.83; $\left(\alpha_{2}\varepsilon_{2}\right)$ = 6.54. Water–kaolinite: $\left(\alpha_{1}\varepsilon_{1}\right)$ = 4.57; $\left(\alpha_{2}\varepsilon_{2}\right)$ = 4.68. Pyridine–montmorillonite: $\left(\alpha_{1}\varepsilon_{1}\right)$ = 6.18; $\left(\alpha_{2}\varepsilon_{2}\right)$ = 6.61.

## Data Availability

The data presented in this study are available on request from the corresponding author.

## Sample Availability

Not available.
